# Thermoregulation for very preterm infants in the delivery room: a narrative review

**DOI:** 10.1038/s41390-023-02902-w

**Published:** 2024-01-22

**Authors:** Emma A. Dunne, Colm P. F. O’Donnell, Britt Nakstad, Lisa K. McCarthy, on behalf of the European Society for Paediatric Research (ESPR) Neonatal Resuscitation Section Writing Group

**Affiliations:** 1 Department of Neonatology, The National Maternity Hospital, Holles Street, Dublin, Ireland; 2 School of Medicine, University College Dublin, Dublin, Ireland; 3 Division of Pediatric and Adolescent Medicine, Institute of Clinical Medicine, University of Oslo, Oslo, Norway; 4 Department of Pediatrics and Adolescent Health, University of Botswana, Gaborone, Botswana

## Abstract

**Abstract:**

Abnormal temperature in preterm infants is associated with increased morbidity and mortality. Infants born prematurely are at risk of abnormal temperature immediately after birth in the delivery room (DR). The World Health Organization (WHO) recommends that the temperature of newly born infants is maintained between 36.5–37.5^o^C after birth. When caring for very preterm infants, the International Liaison Committee on Resuscitation (ILCOR) recommends using a combination of interventions to prevent heat loss. While hypothermia remains prevalent, efforts to prevent it have increased the incidence of hyperthermia, which may also be harmful. Delayed cord clamping (DCC) for preterm infants has been recommended by ILCOR since 2015. Little is known about the effect of timing of DCC on temperature, nor have there been specific recommendations for thermal care before DCC. This review article focuses on the current evidence and recommendations for thermal care in the DR, and considers thermoregulation in the context of emerging interventions and future research directions.

**Impact:**

- Abnormal temperature is common amongst very preterm infants after birth, and is an independent risk factor for mortality.
- The current guidelines recommend a combination of interventions to prevent heat loss after birth. Despite this, abnormal temperature is still a problem, across all climates and economies.
- New and emerging delivery room practice (i.e., delayed cord clamping, mobile resuscitation trolleys, early skin to skin care) may have an effect on infant temperature. This article reviews the current evidence and recommendations, and considers future research directions.

## Introduction

Newly born preterm infants lose body heat rapidly after birth. Abnormal admission temperature is associated with increased mortality and morbidity.^1–16^ The World Health Organization (WHO) and the International Liaison Committee on Resuscitation (ILCOR) recommend that the temperature of newly born infants is maintained between 36.5–37.5 °C, and that hypothermia and hyperthermia are avoided.^17,18^ ILCOR suggest that infants born before 32 weeks’ gestational age (GA) should be cared for under radiant heat (RH) in the delivery room (DR), in addition to a combination of other interventions such as keeping room temperature 23–25 °C, warm blankets, plastic bag/wrap (PB), hat, exothermic mattresses (EM) and heated and humidified (HH) ventilation gases.^17^ The optimal combination of interventions to achieve normothermia in preterm infants is not known. Recent research suggests that there may be a role for placing preterm infants skin to skin after initial stabilization in the DR,^19–23^ with the additional potential benefits of parental bonding, breast milk production and application in low income settings.

Historically, the aim of thermal care interventions in the DR was to prevent hypothermia. Hypothermia is still common in the newborns, even in hot climates, and efforts to reduce hypothermia have been associated with an increase in hyperthermia (>37.5 °C),^3,6,11^ which is thought to be harmful.^6,11,24–27^ In 2015, ILCOR recommended that the umbilical cord be clamped at least 30 s after birth in uncompromised preterm infants.^17^ At present, there are no specific recommendations for temperature management during delayed cord clamping (DCC) or for temperature monitoring in the DR.^28^ This review provides an overview of the current evidence base for thermoregulation in the DR. We consider DR interventions that may affect thermal care, and make recommendations for future research.

## Evolution of thermal care in the delivery room

Hypothermia in preterm infants has been linked to mortality for over 150 years.^29,30^ In 1870, Tarnier was one of the first to describe incubator care to keep preterm infants’ from “succumbing to hypothermia”.^29^ In 1907, Budin wrote that the newly born preterm infant “becomes chilled with astonishing rapidity”, and when they were “admitted with a marked fall of temperature” he could “almost with certainty, foretell their death.”^30^ In the 1950s, Silverman et al. published a series of randomized trials, which demonstrated increased survival in preterm infants cared for in a warmer environment.^31–35^ In the decade that followed, trials focusing on incubator care, room temperature and humidity in the NICU corroborated these results.^36–38^ Baum and Scopes proposed a polyester wrap lined with aluminum foil (“silver swaddler”) to prevent heat loss in term infants.^39^ In 1971, Besch published the results of the first trial focusing on heat preservation in the DR comparing a combination of interventions (RH, hats, and plastic [bubble] wrap). Besch was also the first to describe the heat-preserving properties of polyethylene in newly born term infants.^40^ Following this, a small observational study reported that combining polyethylene wrap with radiant heat reduced oxygen consumption, TEWL and radiant heater output (on servo control).^41^ Twenty-eight years after Besch’s publication, Vohra et al. published the first randomized trial of heat loss prevention in preterm infants in the DR using a PB under radiant heat.^42^ There have since been numerous randomized controlled trials examining the efficacy of different interventions to prevent heat loss after birth (hats, PB, EM, HH gases for respiratory support, RH, increasing room temperature, and servo control).^43–45^ The majority of these studies have reported a positive effect on temperature that have informed a change in practice. All randomized trials on thermal care that inform the current guidelines were performed before DCC became a mainstay of DR management for preterm infants, and the vast majority of interventions were studied under radiant heat.^43^ The effect of the prolonged interval between birth and DCC, and placement of the infant under RH on thermoregulation has yet to be established.

## Definition of temperature ranges for newly born infants

The WHO defines normal temperature as a core temperature between 36.5 °C and 37.5 °C. Temperature outside of this narrow range is associated with morbidity and mortality in newly born preterm infants.^1–6,8–11^ It is therefore recommended that the temperature of newly born preterm infants is maintained between 36.5 °C and 37.5 °C after birth and during stabilization, and the hypothermia and hyperthermia are avoided.^18^

## Epidemiology

Maintaining normal temperature in newly born preterm infants is a global problem^9,11–13,15,25^ and the reported incidence of abnormal temperature varies widely from 5 to 85%.^2,5,8^

In one study, over one-third (36%, *n* = 754/2098) of infants did not have an admission temperature recorded.^11^ The practices at individual sites are often not documented and information that could explain the variation such as site and timing of measurement, device used, and interventions to prevent heat loss are not reported. The definition of hypothermia varies between studies, from <35.0 °C^1^ to 36.0 °C^3,4,46^ to <36.5 °C. Inconsistent definitions of hypothermia make it difficult to compare studies and contribute to considerable variation in the reported incidence of hypothermia.^2,5^

Several large cohort studies describe an inverse relationship between low temperature on admission and neonatal mortality.^1–6,8–11^ A systematic review of prediction models for mortality found that normal temperature on admission is one of eight predictors of survival in preterm infants.^7^ Despite the clear association between hypothermia and mortality it is not clear if the etiology is inherent or iatrogenic.^47^ Reilly et al. conducted the only randomized trial on thermoregulation in preterm infants that was powered to detect a difference in mortality. There was no significant difference in mortality (OR 1.0, 95% CI 0.7–1.5) and the trial was stopped early for futility. The authors speculate that hypothermia may be a marker for death but that a PB does not prevent hypothermia-associated death.^48^ A recent meta-analysis has reported an association between LBW infants with hypothermia on admission and morbidity and mortality; intra ventricular hemorrhage (OR  =  1.86; 1.09–3.14), bronchopulmonary dysplasia (OR  =  1.28; 1.16–1.40), neonatal sepsis (OR = 1.47; 1.09–2.49), retinopathy of prematurity (OR  =  1.45; 1.28–1.72), mortality (OR = 1.83; 1.72–2.09).^49^

A number of recent cohort studies report that the distribution of admission temperature has changed; more infants have a temperature within the normal range, fewer are hypothermic,^1,3,6^ and more are hyperthermic.^3,6,11,27^ This shift is likely due to recognition of hypothermia as a problem and efforts to reduce it in the DR. Hyperthermia occurs less frequently than hypothermia, which is still the prevailing issue. Compared to hypothermia, less is known about the effects of hyperthermia on neonatal outcome, in particular hyperthermia in the absence of infection or inflammation e.g., iatrogenic hyperthermia. The mechanism of harm with hyperthermia is unknown, and may be related to inflammation and/or infection.^50^ Hyperthermia increases metabolic demand and oxygen consumption^26^ and is associated with neuronal injury in the setting of chorioamnionitis.^24–26^ Hyperthermia in preterm infants has been associated with major cranial ultrasound abnormality (OR 1.48, 95% CI 1.11–1.97)^51^ and mortality (OR 1.32, 95% CI 1.11–1.56).^52^ Two large cohort studies have reported a U-shaped relationship between admission temperature and morbidity and mortality.^6,11^ No large cohort studies have reported on adverse outcomes associated with hyperthermia alone.^17^ While the guidelines recommend that hyperthermia is avoided, there is no specific guidance on the management of infants with hyperthermia after birth. Further research is needed in this area.

## Mechanisms of heat loss

Heat is a form of energy that is transferred across a gradient from areas of higher to lower temperature. The temperature of the fetus in utero is ~0.5 °C higher than maternal temperature,^53,54^ and is regulated by placental blood flow and a relatively neutral temperature gradient between the fetus and its surroundings.^53^ At birth the infant is exposed to an ambient temperature 10–15 °C lower than its in-utero environment. This temperature gradient will result in heat loss by conduction (to cooler surfaces), convention (through the circulating air), radiation (to relatively colder surfaces not in direct contact with the infant) and evaporation (from their wet, immature skin).

Preterm infants are particularly vulnerable to heat loss. A high surface area to mass ratio and immature skin deficient in keratin predisposes to high evaporative heat loss.^55^ Trans-epidermal water loss (TEWL) is greatest during the first minutes of life and decreases over the first hour after birth. TEWL increases with decreasing GA. The TEWL of an infant born at 25 weeks’ GA is estimated to be 15 times that of an infant born at term.^56^ A lack of insulating subcutaneous fat and relative inability to vasoconstrict due to poor vasomotor control further exacerbate heat loss.^57^ Additionally, the preterm infant has very limited ability to generate heat.^58^ Without an external heat source maintaining normothermia is a challenge.^31^ Net heat loss will rapidly exceed heat gain, leading to hypothermia. Interventions to prevent hypothermia in newly born infants therefore aim to prevent heat loss and provide external heat.

## International recommendations for thermal care in the delivery room

**Fig. 1:**
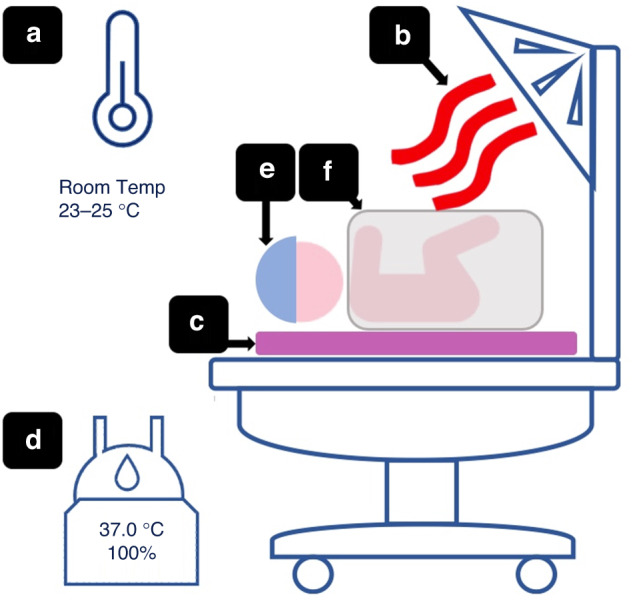
Interventions to improve thremoregulation after birth, in the delivery room. [i] external heat sources (**a** room temperature, **b** radiant warmer, **c** exothermic mattress, **d** heated and humidified gases) and [ii] interventions that prevent heat loss (**e** hat, **f** polyethylene bag).

ILCOR suggest that interventions to maintain normal temperature should be applied within 60 s of birth. Infants born less than 32 weeks’ GA should be cared for under radiant heat in the DR, in addition to using a combination of other interventions which may include; room temperature 23–25^o^C, warm blankets, PB, hat, EM, and HH gases (Fig. 1).^17^ In 2015, ILCOR suggested that cord clamping (CC) is deferred for 60 s after birth in uncompromised preterm infants.^17^ There are no specific suggestions for temperature management between birth and the time of DCC or for temperature monitoring in the DR.^28^ The optimal combination of interventions to achieve normothermia in preterm infants is not known.

## Interventions to improve thermoregulation in the delivery room

### External heat sources

#### Radiant Heat

Preterm infants have limited ability to produce heat. The magnitude of radiative heat loss is determined by the temperature gradient between the infant’s skin and the surfaces adjacent to the infant.^59,60^ One study reported that heat lost through radiation in the delivery room is three times that of an infant in an incubator.^61^ When an infant is placed on the resuscitation trolley with an overhead warmer, the warmer emits radiant heat at a temperature that exceeds that of the infant (38–42 °C). This results in a net heat gain and reduces heat loss by radiation. The contribution of the room temperature to heat loss is limited when an infant is under RH^62^ and wrapping infants in polyethylene permits passage of RH to the infant.^63^ The temperature output of RH varies between commercially available devices, and there is no guidance on the desired target output temperature. Depending on the setting and device, infants may be at risk of hypothermia or hyperthermia.^62^ RH is a mainstay of treatment for preterm infants in the DR and is recommended for all infants <32 weeks GA.^17^ The vast majority of DR trials examining interventions to improve thermoregulation have used a radiant warmer in both the intervention and control arms.^43^ Traditionally, the radiant warmer is located on a resuscitation trolley, away from the mother. Since the introduction of DCC the median time to place an infant on a resuscitation trolley and under radiant heat is now greater than the 60 s interval recommended by ILCOR.^64^ It is not known whether this affects infant temperature. Two randomized trials reported that the time to admission (a surrogate measurement of time under radiant heat) was positively correlated with body temperature, but these results were not statistically significant (*p* = 0.07, *p* = 0.08).^65,66^

Servo-control aims to maintain a normal temperature by continuously monitoring the infant’s skin temperature and adjusting the radiant heat output accordingly.^60^ A multi-center randomized trial compared servo-control in the DR to radiant heat at maximum output in very preterm infants. A similar number were normothermic on admission (39.6% vs 42.2%, *p* = 0.63). Infants in the servo-control arm were at increased risk of mild hypothermia (RR 1.48, 95% CI 1.09–2.01), while none were hyperthermic.^67^ In the DR, RH should be set to maximum output to pre-warm the resuscitation trolley. Infants should be placed under RH at maximum output after birth. Servo control should be considered where time under radiant heat is prolonged (i.e., extensive resuscitation), as these infants may be at risk of iatrogenic hyperthermia.

### Exothermic mattresses

Exothermic mattresses (EM) are sodium acetate gel-filled mattresses that crystallize when activated, producing latent heat. When activated, an EM reaches its maximum temperature of 38 42 °C in 3–5 min. This temperature is sustained for ~1 h.^68^ When an infant with a temperature lower than the EM is placed on it, heat is transferred by conduction from the mattress to the infant. If an infant is placed on a mattress that is not activated, they may lose heat by the same mechanism.^69^ Observational studies have reported that the addition of an EM to radiant heat and a PB is associated with a reduction in the incidence of admission hypothermia (3% [EM + PB] vs 23% [PB])^70^(26% [EM + PB] vs 69% [PB]),^71^ and an increase in admission hyperthermia (50% [EM + PB] vs 30% [PB],^70^ 28% [EM + PB] vs 4% [PB]^71^). Three randomized trials compared PB + EM versus PB alone in the DR in preterm infants. Two reported less hypothermia on admission to the NICU in the intervention group (34% vs 57% [*p* < 0.05],^46^ 41% vs 68% [*p* = 0.10],^72^ and neither found a significant difference in hyperthermia between the groups.^46,72^ A third study reported that more infants in the intervention [PB + EM] group had an admission temperature outside of the normal range (59% vs 23%, *p* = 0.002), largely due to significantly more infants in the intervention group having hyperthermia (46% vs 17%, *p* = 0.009). Based on the recommendations of the safety committee, recruitment ceased after 72/116 infants were enrolled.^65^ The EM is a portable, simple intervention that may prevent heat loss when activated in a timely fashion and used in conjunction with a PB. It poses a risk of hyperthermia^65,70^ and should be used with caution. The use of servo-control with this intervention could reduce the incidence of hyperthermia and warrants further investigation.

### Heated, humidified gases

In the NICU, it is standard practice for gases to be heated and humidified (HH) to 37 °C with 100% relative humidity for infants on respiratory support. This helps to prevent hypothermia and damage to the lung epithelium. The temperature of piped air and gas is 23.3–23.4 °C, with a relative humidity of 2.1–5.5%.^73^ When used in the DR, a large internal surface area of the respiratory tract and lung is exposed to cold.^74^ Using HH gases in the DR may improve infants’ temperature. However, there are some operational barriers. A benchtop study reported that the minimum time to steady state heating and humidification of gases is 8 min,^75^ and an uninterrupted power supply is needed. A prospective cohort study of infants ≤32 weeks’ that received HH gases in the DR reported an increase in admission normothermia (43% vs 12%, *p* < 0.001).^76^ A meta-analysis of two randomized trials comparing HH gases to cold piped gases reported a reduced risk of admission hypothermia in favor of HH gases for infants <28 weeks GA (RR 0.61, 95% CI 0.14–0.73).^77^ The recruitment rate for these trials was 60%^78^ and 63%,^79^ respectively, and infants born to mothers with peripartum pyrexia were excluded.^78,79^

### Room temperature

Reducing the temperature gradient between a newly born infant and its environment should attenuate heat loss. A randomized trial of 96 infants born at <32 weeks’ GA compared DR temperatures of 20–23 °C versus 24–26 °C. Fewer infant in the intervention group (24–26 °C) were hypothermic on admission (68% vs 34%, *p* < 0.01). Neither PB nor EM were used in the DR for this trial.^80^ A randomized trial (*n* = 809, all GA) compared room temperatures (20 °C vs 23 °C) reported no difference in the rate of hypothermia in preterm infants. The authors hypothesize that the use of warming adjuncts (PB, EM) in this subgroup mitigated the effect of room temperature.^81^ An in-vitro study reported that the contribution of room temperature (22–26 °C) to heat loss is reduced when an infant is under RH.^62^ In the era of DCC, infants are exposed to the DR environs before the cord is cut and they are transferred to the resuscitation trolley. The lower the room temperature, the greater the risk of heat loss, and without an external heat source, they will lose heat to their surroundings. Practically, it can be difficult to warm DRs due to limitations of infrastructure and staff comfort. Many hospitals report DR temperatures that are lower than recommended.^5,82^

## Interventions to prevent heat loss

### Polyethylene bags

A single-layer polyethylene wrap can prevent heat loss by evaporation and conduction while allowing the passage of radiant heat to the infant.^63^ A systematic review and meta-analysis of 13 randomized trials (*n* = 1633) reported that using a PB for preterm infants in the DR increases admission temperature (mean difference 0.58 °C, 95% CI 0.50–0.66), reduces hypothermia (RR 0.67, 95% CI 0.62–0.72, NNT = 4) and increases normothermia (RR 0.75, 95% CI 0.69–0.81. NNT 5). Using a PB in the DR confers a risk of hyperthermia (RR 3.91, 95% CI 2.05–7.44, NNT 25).^44^ All but one trial of PBs utilized radiant heat in the control and intervention groups.^43^ The efficacy of a PB without radiant heat in preterm infants is not well described and should be considered in the design of future studies and in the design of mobile resuscitation trolleys.

### Hats/head coverings

A standard hat covers 15.6% of the total body surface area of a term infant.^83^ Heat loss is reduced by 25% in term infants wearing a gamgee hat, compared to infants without hats (1.4 [0.09] °C/h vs 1.9 [0.12] °C/h, *p* < 0.005).^83,84^ A randomized trial (*n* = 80, 24–34 weeks’ GA) reported no difference in admission hypothermia or mean admission temperature between cotton and polyethylene caps (92.5 vs 100%, 35.3 °C vs 35.1 °C, *p* = 0.36).^85^ Another trial (*n* = 100, <29 weeks’ GA) compared total body (including head covering) polyethylene wrap to body alone and found that the proportion of infants with moderate hypothermia (<36.0 °C) was lower in the total body group, however, this difference was not statistically significant (12% vs 20%, *p* = 0.41).^66^ A third study (*n* = 96, <29 weeks GA) reported that infants in polyethylene wraps or polyethylene caps alone maintained their temperature more effectively than infants in the control group that were dried and not placed in any covering.^86^ Since the introduction of DCC, the time to hat placement has increased.^64^ Heat may be lost during this time, and studies investigating the feasibility and efficacy of early hat placement before CC are warranted.

### Immediate skin-to-skin care

Skin to skin care (SSC) was first described over 40 years ago by Rey et al. as a means to maintain temperature in LBW infants born in low income settings, where incubators were scarce.^87^ A systematic review of 30 randomized trials of Immediate skin-to-skin care (I-SSC) identified a paucity of evidence for thermoregulatory stability in very preterm infants.^88^ An observational study of 688 infants reported the duration of SSC is negatively correlated with rectal temperature in infants <38 weeks GA, suggesting the SSC alone is not adequate for thermoregulation in preterm infants.^89^

A small randomized trial of 55 infants demonstrated that I-SSC is feasible in the first hour of life for infants’ 28–34 weeks’ GA. However, the authors identified a risk of hypothermia in this cohort, reporting significantly lower temperatures in the I-SCC group at NICU admission (36.2 °C [0.55] vs 36.5 °C [0.49] *p* = 0.04) and at 1 h (36.3 °C [0.52] vs 36.6 °C [0.42], *p* = 0.03).^20^ Lode-Kolz et al. randomized 91 infants (28–33 weeks GA) to I-SCC versus conventional care in the first 6 h of life. The incidence of hypothermia did not differ significantly between the groups (15/46 vs 12/45, RR [95% CI] 1.2 [0.65, 2.32], *p* = 0.54). The median (IQR) time to initiate I-SCC in the lower GA strata (28–31 + 6 weeks GA) was 1 (0.5–1.3) h(s).^23^ This may point to some logistical obstacles for true I-SCC (i.e., immediately after birth, in the delivery room). I-SCC has demonstrated a reduction in mortality for LBW infants in low-income settings.^19^ It is an affordable and widely available resource. An ILCOR commissioned systematic review of thermal care in the delivery room identified a paucity of high-quality evidence for SCC maintaining normal temperature after birth and has identified this area as a research priority, especially for low-resourced settings.^45^

## Delivery room interventions that may affect thermoregulation

### Cord clamping

Since 2015, ILCOR has suggested waiting for at least 30–60 s before CC in uncompromised preterm infants.^17^ In this case, there is potentially a period of at least 30 s in which the naked wet, preterm infant, with immature skin and a high surface area to body mass ratio is exposed to the DR environs. Their ability to generate heat is limited. Without an external heat source, infants may lose heat through evaporation, radiation, and convection. A study of near-term lambs reported that thermoregulation is maintained during physiological-based cord clamping (PBCC). The authors suggest that temperature is maintained by placental blood flow during DCC, however, measures were taken to prevent heat loss before CC (drying, hot water bottles, blankets, hair dryers), making it difficult to draw definitive conclusions about the influence of placental blood flow on infant temperature after birth.^90^ Randomized trials comparing delayed versus immediate cord clamping (ICC) have reported no significant difference in mean admission temperature between groups.^91^ All of these studies reported temperature as a secondary outcome and refer only to mean temperature difference. These studies do not report the distribution of temperature or the number of infants with temperature outside of the normal range. The majority of published RCTs examining interventions to prevent hypothermia in preterm infants in the DR immediate cord clamping (ICC) was routine. Infants were promptly transferred to the resuscitation trolley hence, measures taken to prevent heat loss and provide warmth were initiated almost immediately after birth. Importantly, almost all trials utilize RH in both the intervention and control arms.^43^ In a small prospective cohort study of infants undergoing DCC (*n* = 54), the authors noted an increase in the incidence of hypothermia compared to a similar cohort at their hospital before DCC was introduced (54%^92^ vs 6%^65^). Since the introduction of DCC, the median time to initiate thermal care interventions (place under RH, hat, PB) is greater than the 60 s recommended by ILCOR.^64^ To the best of our knowledge, there are no published studies focusing on thermal care before CC, and there are no recommendations in the resuscitation guidelines for thermal care during DCC. A recent systematic review and meta-analysis of interventions to prevent hypothermia in infants <34 weeks GA reported that the optimal methods for maintaining normal temperature during DCC could not be define because too few studies reported their approach to cord management or temperature outcomes after DCC.^45^

### Resuscitation trolleys

Recently the focus of cord clamping has shifted to PBCC.^93–100^ Providing resuscitation with an intact cord to those infants that are not breathing spontaneously is a rapidly evolving topic in DR management, and there is a tendency towards increased time to CC. A number of mobile resuscitation trolleys (MRT) have been manufactured and studies of feasibility and efficacy are ongoing.^97,101^ The thermal care afforded by commercially available MRTs varies from none, to a heated mattress (e.g., LifeStart^TM^) or an overhead radiant heater (e.g., Concord™). In a study comparing ICC (<20 s) or placement on an MRT with CC at 2 min there was no difference in the incidence of admission hypothermia between the two groups (13% vs 14%),^102^ however the authors did not describe the thermal care provided for either group. Pratesi et al. randomized 40 infants born <30 weeks’ GA to intact cord stabilization for 3 min on the LifeStart^TM^ MRT (Inspiration Healthcare) compared to cord milking and clamping before 20 s. All infants were placed in a PB after birth. The LifeStart^TM^ MRT has an in-built warming mattress with a maximum output of 40 °C, which activates when the infant is placed on it. The mean (SD) admission temperature for the MRT group was significantly lower than the early cord clamping groups (35.5 [0.7] vs 36.0 [0.7] °C, *p* < 0.01). The authors commented that the DR temperature (22–24 °C) may have contributed to this difference, since the LifeStart^TM^ MRT does not have an overhead radiant warmer. Additionally, some infants may have been too light to activate the warming mattress.^103^ A randomized trial (*n* = 150 infants, mean GA 28 weeks) compared ventilation on an MRT during DCC (V-DCC) to DCC and tactile stimulation alone. None of the infants were under radiant heat before CC, however, the infants assigned to V-DCC were placed on an exothermic mattress during the intervention, while the infants in the DCC group were not. Even though one group was exposed to an external heat source, there was no difference in the mean admission temperature between the two groups (mean difference 0.02 °C, *p* = 0.975).^104^

Brouwer et al. performed a feasibility study of the stabilization of 37 infants < 35 weeks’ GA with intact cord using an MRT with an overhead radiant warmer (Concord™). They reported a high rate of admission hypothermia in their cohort (60% < 36.5 °C), but state that this is comparable to their baseline incidence of hypothermia at their hospital.^94^ These findings require careful consideration when implementing MRT’s to stabilize preterm infants in the DR, as additional warming adjuncts and regular temperature monitoring may be required to maintain infant normothermia.

## Conclusion

Abnormal temperature on admission to the NICU is a global problem, and is associated with morbidity and mortality in preterm infants. Current guidelines recommend using a combination of interventions in the DR to prevent heat loss. Despite this admission hypothermia is still prevalent, with hyperthermia also increasing. The landscape of the DR is evolving, and thermal management needs to keep up. Immediate after birth skin-to-skin care is a promising thermoregulatory alternative, that can be applied in all settings. Since the widespread introduction of DCC and a move towards PBCC, there have been no new recommendations for thermal care. Pragmatic, well-designed prospective studies examining the effect of deferred cord clamping and mobile resuscitation trolleys on temperature control are needed. Further rigorous examination of interventions to prevent abnormal temperature is also warranted.

## Data Availability

Data sharing is not applicable to this article as no datasets were generated or analyzed during the current study.
